# MicroRNA cerebrospinal fluid profile during the early brain injury period as a biomarker in subarachnoid hemorrhage patients

**DOI:** 10.3389/fncel.2022.1016814

**Published:** 2022-11-25

**Authors:** Leire Pedrosa, Jhon Hoyos, Luis Reyes, Laura Llull, Daniel Santana, Nicolás de Riva, Ricard Mellado, Xavier Sala, Ana Rodríguez-Hernández, Joaquim Enseñat, Sergio Amaro, Ramon Torné

**Affiliations:** ^1^August Pi i Sunyer Biomedical Research Institute (IDIBAPS), Barcelona, Spain; ^2^Department of Neurosurgery, Institute of Neuroscience, Hospital Clinic of Barcelona, Barcelona, Spain; ^3^Comprehensive Stroke Center, Institute of Neuroscience, Hospital Clinic of Barcelona, Barcelona, Spain; ^4^Neuroanesthesia Division, Department of Anesthesiology, Hospital Clinic of Barcelona, Barcelona, Spain; ^5^Department of Anesthesiology and Critical Care, Hospital Clínic of Barcelona, Barcelona, Spain; ^6^Department of Neurosurgery, Germans Trias i Pujol University Hospital, Barcelona, Spain; ^7^Department of Medicine, Faculty of Medicine and Health Sciences, University of Barcelona, Barcelona, Spain

**Keywords:** microRNA, subarachnoid hemorrhage, biomarkers, early brain injury, delayed cerebral ischemia, neurovascular, NanoString

## Abstract

**Introduction:**

Delayed cerebral ischemia (DCI) is a dreadful complication present in up to 30% of patients with spontaneous subarachnoid hemorrhage (SAH). Indeed, DCI is one of the main causes of long-term disability in SAH, yet its prediction and prevention are troublesome in poor-grade SAH cases. In this prospective study, we explored the potential role of micro ribonucleic acid (microRNA, abbreviated miRNAs)—small non-coding RNAs involved in clue gene regulation at the post-transcriptional level—as biomarkers of neurological outcomes in SAH patients.

**Methods:**

We analyzed the expression of several miRNAs present in the cerebrospinal fluid (CSF) of SAH patients during the early stage of the disease (third-day post-hemorrhage). NanoString Technologies were used for the characterization of the CSF samples.

**Results:**

We found an overexpression of miRNAs in the acute stage of 57 SAH in comparison with 10 non-SAH controls. Moreover, a differential expression of specific miRNAs was detected according to the severity of clinical onset, but also regarding the development of DCI and the midterm functional outcomes.

**Conclusion:**

These observations reinforce the potential utility of miRNAs as prognostic and diagnostic biomarkers in SAH patients. In addition, the identification of specific miRNAs related to SAH evolution might provide insights into their regulatory functions of pathophysiological pathways, such as the TGF-β inflammatory pathway and blood-brain barrier disruption.

## Introduction

Spontaneous subarachnoid hemorrhage (SAH) is a clinical syndrome associated with high initial morbidity and mortality. The incidence of SAH in the general population is about 10 per 100,000 individuals annually and represents 5–7% of the total incidence of stroke (Vivancos et al., 2014). An underlying ruptured intracranial aneurysm is by far the most common cause of spontaneous SAH (around 80–85% of cases) (Schertz et al., 2016). A very small -almost anecdotal- subset of SAH cases may be the result of other vascular lesions such as arteriovenous malformations or dural arteriovenous fistulas. The remaining 15–20% are usually considered idiopathic SAH cases after the corresponding diagnostic investigations have ruled out a subjacent vascular lesion.

Regardless of the underlying cause, a major prognostic determinant of long-term disability in SAH patients is the occurrence of delayed cerebral ischemia (DCI) (Vergouwen et al., 2010). DCI is defined as a new neurological deficit or decline in the level of consciousness not attributed to the aneurysm’s occlusion treatment or to other non-ischemic causes, occurring at least 48 h after SAH onset (Oka et al., 2019). This complication occurs in up to 17–30% of SAH patients, typically starts on days 4–7 after the initial hemorrhage (Schmidt et al., 2007; Ionita et al., 2010; Rostami et al., 2017) and, as aforementioned, is a well-established and relevant clinical surrogate marker for neurological outcomes after SAH (Vergouwen et al., 2010). Numerous factors including age, initial neurological impairment, intraventricular hemorrhage, SAH load, and aneurysm size, are associated with the development of DCI (Kassell et al., 1990; Rabinstein et al., 2004; Sughrue et al., 2011). Interestingly, recent research suggests that DCI may be in fact a consequence of the amount of early brain injury (EBI) occurring within the first 72 h after SAH (Flynn and Andrews, 2015; Topkoru et al., 2017). The pathological changes and mechanisms of EBI are closely related to the severity of the initial hemorrhage and include increased intracranial pressure (ICP), oxidative stress (OS), neuroinflammation, blood–brain barrier (BBB) disruption, brain edema, and cell death (Cahill et al., 2006; Rowland et al., 2012; Shao et al., 2020). Considering that this early brain damage phase could mediate the emergence of DCI, any biological marker for EBI could help identify those SAH patients at higher risk of later neurological decline. Being able to reliably identify in the first few days those patients at higher risk of presenting DCI, could be paramount to improve clinical outcomes in SAH. However, no robust blood or cerebrospinal fluid (CSF) biomarkers of EBI or DCI are readily available yet.

Recently, microRNAs (miRNAs) have emerged as promising biomarkers in the diagnosis of various neurological diseases such as stroke, Parkinson’s disease, traumatic brain injury (TBI), and Alzheimer’s disease. MiRNAs are a family of non-coding RNAs of 17–24 nucleotides that regulate the expression of several target genes at the post-transcriptional level (Lai et al., 2015; Iranifar et al., 2018; Roitbak, 2018). They act intracellularly but are transported outside the cells in exosomes and exist in stable forms in body fluids (Cortez et al., 2011). A few recent studies have measured expression levels of miRNAs in blood and in CSF and have investigated its changes during the EBI period in SAH (Bache et al., 2017; Sheng et al., 2018; Sun et al., 2019). However, little is known yet about these intriguing biomarkers. Understanding their relationship with DCI incidence could become an extremely valuable clinical tool. In an attempt to generate further knowledge on this promising field, the present explorative study aimed to determine the correlation of miRNA levels and miRNA profiles measured in CSF on day three after SAH, with the risk of developing DCI and worse midterm functional outcomes in SAH sufferers.

## Methodology

The study protocol was approved by the institution’s Clinical Research Ethics Committee (HCB/2019/0930) *ad hoc* with national legislation in the field of biomedical research, the protection of personal data (15/1999) and the standards of Good Clinical Practice, as well as complying with the Helsinki Declaration (1975 and 1983 revisions). The patients or their legal representatives signed a consent form before inclusion in the study. Patient records were anonymized before analysis. The study complies with STROBE guidelines for reporting observational studies.

### Patients

#### Observational cohort

**FIGURE 1:**
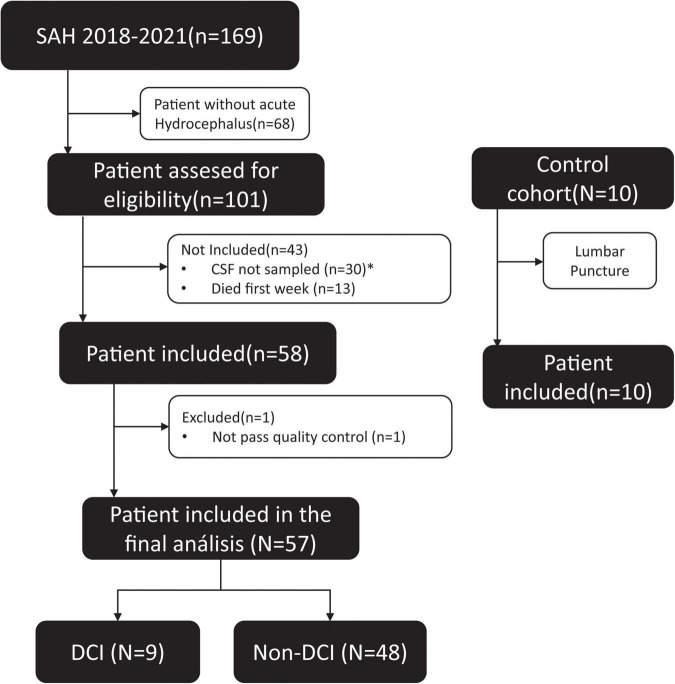
Clinical algorithm of aSAH patients included in the study. *The amount of sample was not sufficient or had not enough quality to extract the RNA, according to the protocol.

A neurovascular database was constructed with prospectively collected data on patients diagnosed with spontaneous SAH at our institution from January 2018 to December 2021 (Figure 1). The analysis included all adult patients (age > 18 years) with the diagnosis of SAH and associated hydrocephalus in whom an external ventricular drainage catheter (EVD) was inserted according to the neurosurgeon’s evaluation. Patients were excluded if death occurred within the first week (Figure 1).

#### Control cohort

A control cohort was used to compare the levels of miRNA in CSF. The control cohort consisted of ten consecutive non-neurological patients, in whom a lumbar puncture was performed as part of the subarachnoid anesthesia procedure prior to a lower-limb orthopedic surgery. These subjects did not suffer from any neurological disease and they signed an informed consent form before being included in the study.

#### Evaluation protocol and sampling schedule

Upon admission to the emergency department, the neurological state was recorded according to the World Federation of Neurological Surgeons (WFNS) grading scale (Teasdale et al., 1988). After EVD insertion, a CSF sample was obtained on day three after SAH onset.

Patients were admitted to either the Surgical Intensive Care Unit (S-ICU) or the Stroke Unit (SU), depending on their clinical status. Neuromonitoring included daily neurological examination [National Institutes of Health Stroke Scale (NIHSS)], daily transcranial Doppler evaluation and daily blood tests. In case a worsening in the neurological situation was suspected, the patient would undergo a basal and a perfusion CT scan.

Upon discharge, patients were visited for midterm follow-up on day 90 after the SAH onset. This final evaluation included a neurological examination and functional evaluation. Functional outcome was assessed with the modified Rankin Scale score (mRS) at 3 months though in-person visits as part of clinical routine and following structured questionnaires.

#### Definition of vasospasm and delayed cerebral ischemia

Radiological vasospasm was defined as the presence of a new-onset narrowing of a vessel, documented in either angio-CT or digital-subtraction angiography (DSA) (De Rooij et al., 2013). DCI was defined as a worsening of at least 2 points compared to the reference score, on the GSC or NIHSS, lasting for at least 2 h, which cannot be entirely attributed to causes other than cerebral vasospasm. Subjects of the observational cohort were divided into DCI and non-DCI patients according to it. The adjudication of vasospasm and DCI was performed by investigators blinded to miRNA data.

### Sampling of cerebrospinal fluid

Using a strict protocol, including sterile precautions, CSF was sampled from the EVD on day 3 after SAH (SAH samples) and before administration of subarachnoid anesthesia for orthopedic surgery in neurologically healthy patients (control samples). All CSF samples were immediately stored on ice and spun within 15 min at 500 *g* for 10 min; the supernatant was stored at –80°C until use.

### RNA extraction

Total RNA was isolated from 200 μl of each sample of CSF according to the manufacturer’s protocol (Total RNA isolation kit, Appendix B, Cat. No. 17200; Norgen Biotek, Thorold, Canada). MS2 carrier RNA (Cat. No. 10165948001, Roche, Switzerland) and synthetic spike-in control oligonucleotide ath-miR159a [Sequence: rUrUrU rGrGrA rUrUrG rArArG rGrGrA rGrCrU rCrUrA, Integrated DNA Technologies (IDT), USA] and osa-miR414 (Sequence: rUrCrA rUrCrC rUrCrA rUrCrA rUrCrA rUrCrG rUrCrC, IDT) were added according to the protocol to improve the miRNA extraction and evaluate the efficacy of the process.

### nCounter and data normalization

Three microliters of extracted RNA were processed according to the manufacturer’s recommendations for the nCounter Human v3 miRNA Expression Assay Kit (CSO-MIR3-12, NanoString Technologies, Seattle). Each sample was scanned for 555 FOV on the nCounter Digital Analyzer (NanoString Technologies). Quality control, normalization, and data analysis were performed using nSolver v4.0 Analysis Software (NanoString Technologies). For each gene, count data was processed as follows: (a) normalization to the geometric mean of both spikes (reference genes), (b) geometric mean of negative control, and (c) considering only the miRNA with more of the negative mean and which were express over this threshold in >20% of all samples.

### Networking analysis

MiRnet v2.0,^1^ a miRNA-centric network visual analytics platform, was used to visualize the predicted interactions between differentially-expressed miRNAs (between DCI groups) and target-genes. Degree cut-off used was 1.0. MiRnet considers the miRNA target gene data collected from four well-annotated databases: miRTarBase v8.0 (Huang et al., 2020), TarBase v8.0 (Karagkouni et al., 2018), and miRecords v1.0 (Xiao et al., 2009). For statistical analysis, edgeR v3.38.4 (Robinson et al., 2010), limma v3.52.3 (Ritchie et al., 2015), and HTqPCR v1.50.0 (Dvinge and Bertone, 2009) have been used from R packages, while for network visualization has been used the jquery v0.1.4 (Sievert et al., 2021), sigma.js v0.1.5 (Coene, 2018), and igraph v1.2.6 (Csárdi and Nepusz, 2006) R packages.

### Pathway analysis

The MIENTURNET (MicroRNA ENrichment TURned NETwork, Release 3.4.4, March 2018)^2^ web tool implemented by using the R programming language^3^ was used to predict the miRNA-target interactions and performed the enrichment analysis (Licursi et al., 2019). MIENTURNET considers both the computational and experimental evidences from TargetScan v7.2 (Agarwal et al., 2015) and miRTarBase v7.0 (Hsu et al., 2011) databases, respectively, to predict the interaction of miRNA on the target gene. To create the enrichment analysis, we selected the miRNAs with two or more miRNA-target interactions and a threshold for the adjusted *p*-value > 1 to both TarScan and miRTarBase. Adjusted p-value was obtained by false discovery rate (FDR) correction, which is the rate that features called significant are truly null or the ratio of the number of false positive results to the number of total positive test results. FDR is a measure of accuracy when multiple hypotheses are being tested at once. Functional enrichment analysis was performed with target genes of selected miRNAs using the Kyoto Encyclopedia of Genes and Genomes (KEGG) and Reactome databases. Functional enrichment analysis (also called gene set enrichment analysis (GSEA) or pathway enrichment analysis) is a method to identify genes or proteins that are over-represented in a set of genes or proteins, and may have an association with disease phenotypes or may be involved in same biological pathway or by proximal location on a chromosome (Subramanian et al., 2005).

### Statistical analysis

#### Clinical data analysis

Continuous variables were reported as mean with standard deviations (SD) or median with interquartile ranges (IQR) and were compared with the Student’s *t*-test, Mann–Whitney, or Kruskal-Wallis tests as appropriate. Categorical variables were compared with the Chi-square and Fisher exact tests. Univariate analysis was used to evaluate the clinical and radiological variables associated with DCI. The analysis was performed using SPSS v26.0 (IBM SPSS Statistics for Windows, Armonk, NY, USA), and the level of significance was established at a 0.05 level (two-sided).

#### Laboratory data analysis

All analyses used log_10_-transformed data. The heatmaps, volcano plots and boxplots have been represented by MetaboAnalyst 5.0^4^ (Xia et al., 2009). The normalized expression data were mean-centered and scaled by subtracting the mean and dividing by the standard deviation of each gene across all samples. The Ward method and Euclidean distance were applied to establish optimal hierarchical clustering, which begins with each sample considered as a separate cluster and then proceeds to combine them until all samples belong to one cluster. The features were used to scale and adjust the final heatmap to focus on patterns from important features. A two-tailed unpaired t-student test was performed to compare the expression of genes between two groups. The raw *p*-value and the FDR value (*q*-value or adjusted *p*-value), to correct the *p*-value for multiple comparisons, were calculated.

Principal component analysis (PCA) was performed with the normalized and log_10_-transformed data by MetaboAnalyst 5.0. PCA is an unsupervised method aiming to find the directions that best explain the variance in a data set without referring to class labels. The PCA analysis was performed using the prcomp package v2 (Sigg and Buhmann, 2008) from the R package (R Commander version 4.1.3, Vienna, Austria). The calculation was based on singular value decomposition (SVD).

To compare the predictive effectiveness of each selected miRNA for each group, a receiver operating characteristic (ROC) curve was drawn by R Commander version 4.1.3 with OptimalCutpoints R package v1.1-4 (López-Ratón et al., 2014). The Youden index pointed out the optimal cut-off value to predict the classification of patients to each of the compared groups. The *p*-value was obtained by SPSS for each ROC curve.

## Results

### Patient baseline features

Between 2018 and 2021, a total of 169 patients were admitted to our institution with the diagnosis of spontaneous SAH; of those, 101 (60%) had concomitant acute hydrocephalus requiring an EVD placement. According to the inclusion/exclusion criteria, 57 subjects were finally eligible to be enrolled in the study (Figure 1). Table 1 summarizes the clinical and radiological presentation and the evolution of the included cohort according to the incidence of DCI. Both the DCI and non-DCI group were balanced in demographic terms; the mean age was 57 (±13) years and patients were predominantly females (65%). We found no difference in the amount of initial cisternal blood between DCI and non-DCI patients. All patients had a modified Fisher scale greater than three (Claassen et al., 2001).

**TABLE 1:** Demographic and clinical variables of the aneurysmatic SAH cohort. Data are first described in the global group and then categorized according to the presence or absence of delayed cerebral ischemia (DCI).

	Global (*n* = 57)	DCI (*n* = 9)	No DCI (*n* = 48)	*P*-Value (DCI vs non-DCI)
Age (years), mean (SD)	57 (13)	49 (15)	60 (12)	0.084
Sex (female), *n* (%)	38 (67)	6 (67)	33 (69)	0.639
Hypertension	33 (58.9)	5 (55.6)	28 (60)	0.822
Obesity	8 (14.5)	1 (12)	7 (15)	0.859
Smoking	21 (39.62)	4 (50)	17 (38)	0.515
Excessive alcohol	5 (9.6)	2 (25)	4 (9)	0.195
Consumption of drugs	4 (7.5)	0 (0)	4 (9)	0.380
WFNS, *n* (%)				0.441
1–3	31 (54)	4 (44)	27 (56)	
4–5	26 (46)	5 (56)	21 (44)	
Modified Fisher grade, *n* (%)				0.730
1	0	0	0	
2	0	0	0	
3	4 (7)	1 (11)	3 (6)	
4	53 (93)	8 (89)	45 (94)	
Aneurysm location, *n* (%)				0.432
No aneurism	4 (7)	0	4 (8)	
Anterior circulation	41(72)	8 (89)	33 (69)	
Posterior circulation	12(21)	1 (11)	11(23)	
Aneurysm treatment, *n* (%)				0.354
Embolization	38 (67)	6 (67)	32 (67)	
Clipping	12 (21)	3 (33)	9 (19)	
No treatment	7 (12)	0	7 (14)	
Angiographic vasospasm, *n* (%)	39 (68)	9 (100)	30 (63)	0.024
VP shunt, *n* (%)	31 (54)	8 (89)	23 (48)	0.107
Death at 3-months, *n* (%)	10 (18)	0	10 (21)	0.288
mRS at 3-months, *n* (%)				0.022
0–2	31 (54)	3 (33)	28 (58)	
3–5	16 (28)	6 (67)	10 (22)	

### Clinical outcomes

Overall, nine (16%) of the 57 patients included in the study developed DCI. Angiographic vasospasm was found in 39/57 (67%) patients, this was more frequent within the DCI group than in the non-DCI group (100% vs. 63%, *p* = 0.024). Before discharge, 31 (54%) patients required surgery for ventriculoperitoneal shunting.

At three-month follow-up, a total of 10 (18%) patients were dead. Mortality occurred at a mean time point of 10 days after the hemorrhagic event (range 7–19), and it was higher in the non-DCI group compared to the DCI group (21% vs. 0%). However, functional outcomes at three months were worse in the DCI group. Only 3 patients (33%) from the DCI group achieved a good clinical outcome at 3 months (mRS ≤ 2) compared with 28 patients (58%) from the non-DCI group (*p* = 0.022).

### Expression of microRNAs in subarachnoid hemorrhage vs non-subarachnoid hemorrhage cerebrospinal fluid samples

First of all, we compared the expression of miRNAs between control (*n* = 10) and SAH-CSF samples (*n* = 57). Most miRNAs were found to be overexpressed in SAH patients compared with healthy individuals. After clustering the CSF samples according to the miRNA expression, we observed that most of the controls were in the cluster with low expression of miRNAs, whereas SAH samples were in clusters with high and medium expression. Even so, when comparing the expression of each specific miRNAs, most miRNAs were statistically overexpressed in SAH patients compared with healthy subjects [Fold change (FC) > 2; *p* < 0.01; *q* < 0.01] (Supplementary Table 1 and Supplementary Figure 1). In addition, a PCA analysis clustered the controls and SAH patients in different groups (Supplementary Figure 2).

### MicroRNAs expression according to delayed cerebral ischemia status

**FIGURE 2:**
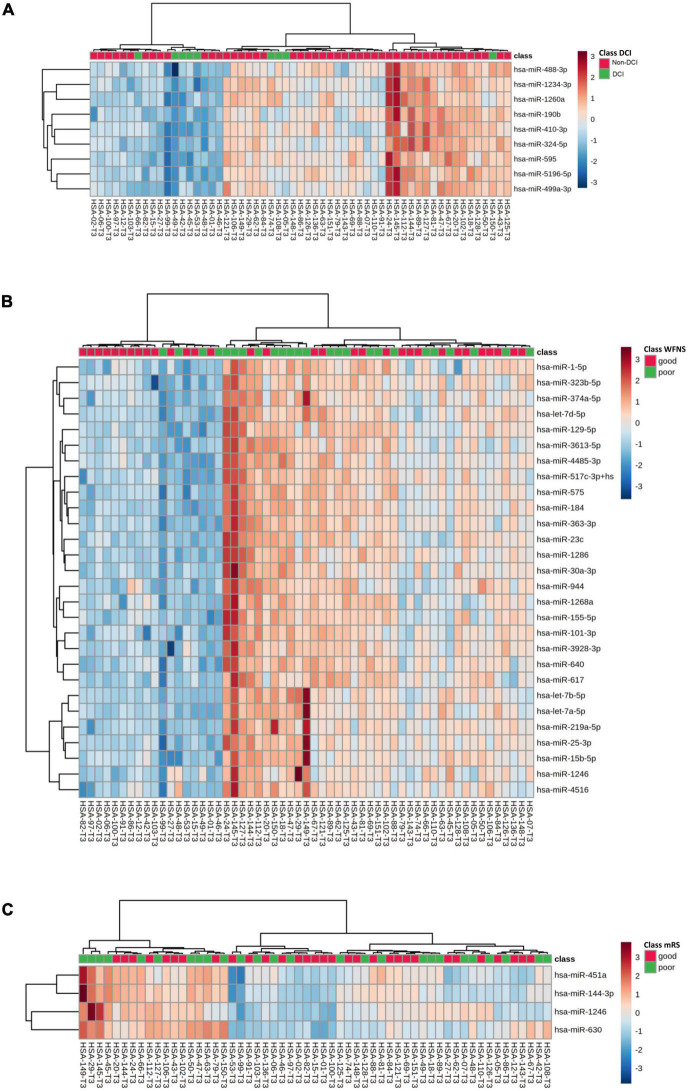
Unsupervised hierarchical clustering analysis obtained with the expression of selected miRNAs in DCI **(A)**, WFNS **(B)**, and modified Rankin Scale at 3 months **(C)** groups. Genes and samples were clustered according to their expression and similarity. On the top, the type of samples is color-coded: green for SAH samples and red for control samples. The color scheme represents the Z-score distribution from –3 (blue, low expression) to 3 (red, high expression).

**FIGURE 3:**
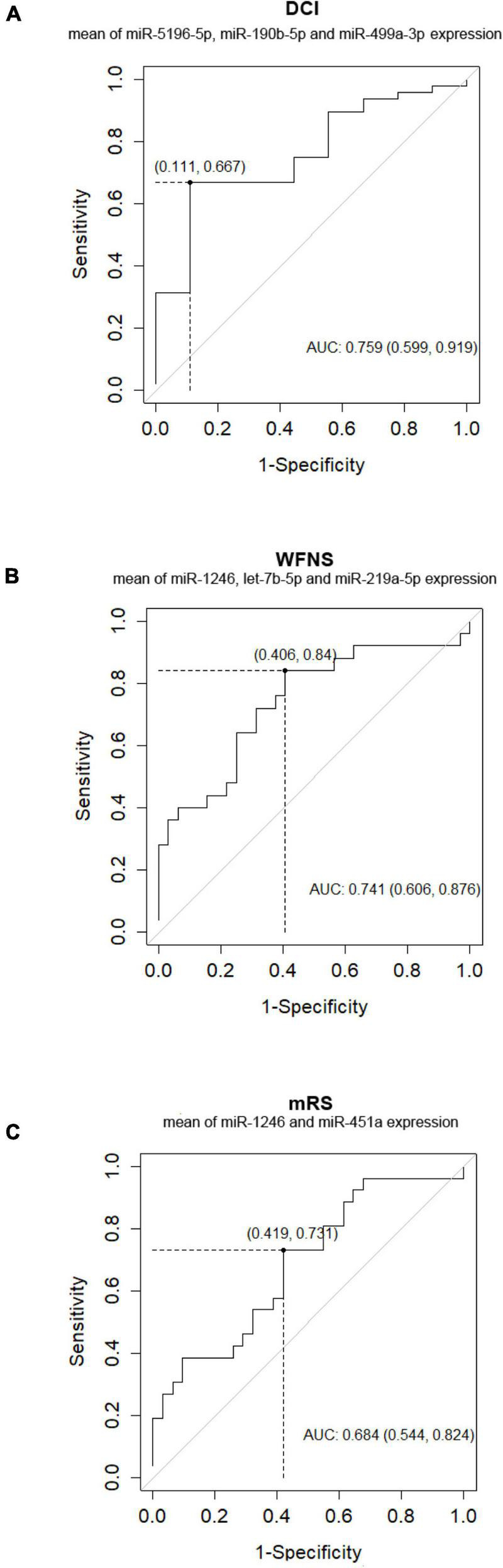
Receiver operating characteristic (ROC) analysis curve for DCI **(A)**, WFNS **(B)**, and outcome at 3 months **(C)** prediction scoring system by mean of few miRNAs.

Out of the 57 SAH patients, 9 (16%) presented DCI during the follow-up. Nine miRNAs were differentially expressed in patients with DCI (*p*-value < 0.05), although significant differences could not be demonstrated after correction for multiple comparisons (*q*-value = 0.650) (Table 2). In addition, 3 of these 9 miRNAs were overexpressed with FC > 2 in non-DCI patients compared with DCI patients (Table 2 and Figure 2A). ROC analysis was performed to study the specificity and the sensibility of each miRNA and of the mean of a panel of selected miRNAs to predict the occurrence of DCI. hsa-miR-190b-5p, hsa-miR-5196-5p, and hsa-miR-499a-3p had a AUC > 0.7 with a *p*-value < 0.05 (Supplementary Table 2). However, these results improved when the mean of these three miRNAs were included in the model (Supplementary Table 2 and Figure 3A).

**TABLE 2:** miRNAs differentially expressed comparing non-DCI vs. DCI.

miRNA	FC	*p*-value	*q*-value
hsa-miR-190b-5p	2.4239	0.013526	0.65033
hsa-miR-5196-5p	2.0583	0.02807	0.65033
hsa-miR-499a-3p	1.9573	0.032308	0.65033
hsa-miR-595	1.8149	0.032399	0.65033
hsa-miR-410-3p	1.8554	0.035793	0.65033
hsa-miR-324-5p	2.2894	0.035988	0.65033
hsa-miR-488-3p	1.6636	0.040428	0.65033
hsa-miR-1234-3p	1.759	0.041671	0.65033
hsa-miR-1260a	1.7558	0.049856	0.65033

The table shows the miRNA with their Fold Change (FC), *p*-value (raw *p*-value), and the *p*-value corrected by FDR (*q*-value). T-test student was performed to obtain the raw *p*-value and FDR correction to obtain the *p*-value adjusted (*q*-value).

### Gene interactions and pathophysiological pathways

**FIGURE 4:**
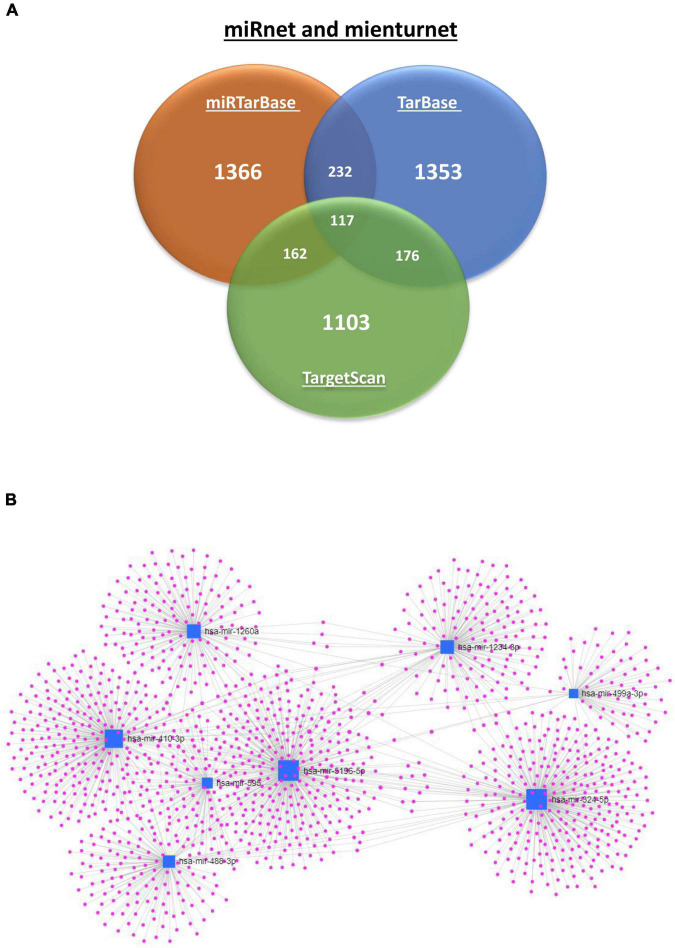
Interaction of 9 miRNA differentially expressed according to DCI occurrence with genes. **(A)** Venn diagram of predicted and validated number of interactions of miRNA with genes by miRTarBase (Orange), TarBase (blue), and TargetScan (green) databases. **(B)** Network analysis was performed with target genes of selected miRNAs of the DCI comparison using miRTarBase database. The miRNAs are represented by a blue nodule and the genes that interact with the miRNAs in purple. The gene interaction is represented by a blue line.

**FIGURE 5:**
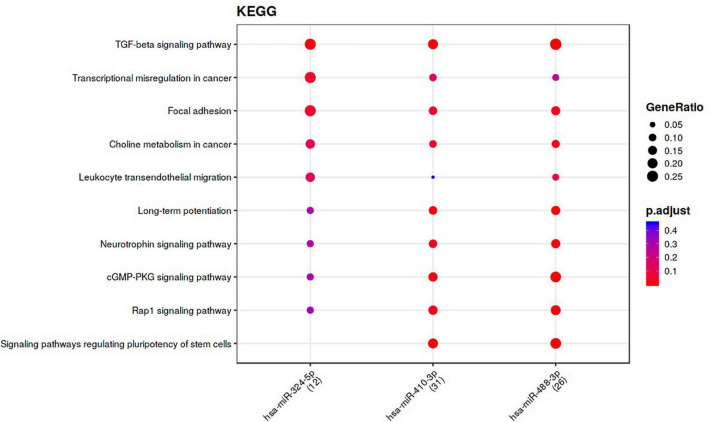
Functional enrichment analysis performed with target genes of the most relevant miRNAs among 9 miRNAs of the DCI comparison, from Target Gene database, using KEGG pathways. The specific miRNAs are shown in x-axes and the pathways obtained with the genes that have interactions with the selected miRNA are shown in the y-axes. The gene ratio is represented by the size of the dot and the color indicates the p-value adjusted by FDR.

The nine differentially-expressed miRNAs in DCI patients were investigated for a possible interaction with specific genes through miRnet and mienturnet platforms. Using both platforms, 1,366, 1,353, and 1,103 gene–miRNAs interactions were assessed with miRTarBase, TarBase, and TargetScan databases. Several genes are shared in two or more databases (Figure 4A). Next, network analyses were performed to visualize and analyze the miRNA-gene interactions (Figure 4B) by miRnet. Then, we selected miRNAs with more than two miRNAs–target gene interactions and an FDR > 1. After applying these filters, three and eight of nine differentially-expressed miRNAs from TargetScan and miRTarBase, respectively, were selected and were used for the functional enrichment analysis. This analysis provided different pathways that might be regulated by the miRNAs, according to the predicted interaction with the target genes. Functional enrichment analysis was performed with the target genes of selected miRNAs using the KEGG and REACTOME databases (Figure 5 and Supplementary Figures 3, 4). Some of the pathways were found to be statistically related to the target genes that interact with the selected miRNAs were TGF-β pathway, focal adhesion and RAP1 signaling, which is involved in adhesion regulation (Figure 5 and Supplementary Figure 3).

### MicroRNAs according to initial clinical severity and midterm clinical outcome

**FIGURE 6:**
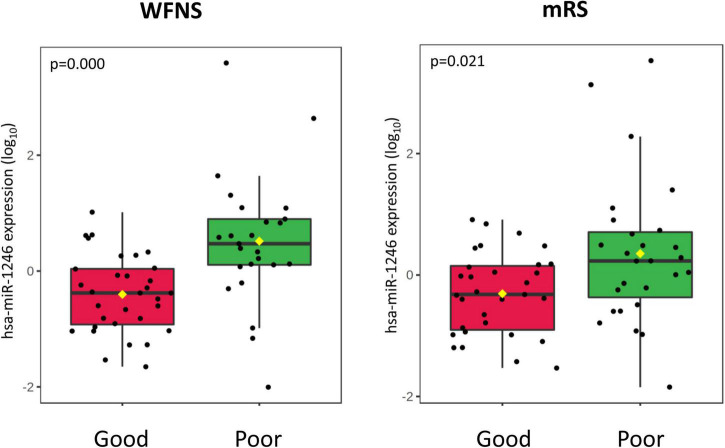
Boxplot of the hsa-miR-1264 miRNA expression according to WFNS at hospital admission **(left panel)** and clinical outcome at 3 months **(right panel)** after SAH.

In our sample, miRNAs expression varied according to initial clinical severity, in-hospital evolution and midterm outcomes. Regarding SAH severity (defined as higher WFNS scores upon admission), 28 miRNAs were differentially expressed between patients with poor neurological presentation (WFNS 4–5) compared with those with a good clinical presentation (WFNS 1–3) (*p* < 0.05, *q*-value > 0.05) (Table 3 and Figure 2B). We also studied the relation between the acute expression of miRNAs at the end of the EBI period and midterm outcomes at 3 months. In this regard, four miRNAs were differentially expressed (Table 4 and Figure 2C). Interestingly, hsa-miR-1246 miRNA was more expressed both in patients with a poor neurological status at admission and those with a poor functional outcome (*p*-value < 0.05) (Figure 6). In addition, the expression of miRNAs, that were differentially expressed in the WFNS comparison (from Table 3), was studied in the mRS groups. We observed that most of the miRNAs overexpressed in the WFNS’ poor group were also more expressed in the poor functional outcome group than in the good clinical outcome group at 3 months. Similarly, the expression of miRNAs that were significantly overexpressed in the subgroup of patients with poor outcomes at three months (from Table 4) tended also to be more expressed in the subgroup of patients with poor WFNS grade at hospital admission, with a *p*-value > 0.05 (Supplementary Figure 5).

**TABLE 3:** miRNAs differentially expressed according to WFNS grade at hospital admission.

miRNA	FC	*p*-value	*q*-value
hsa-miR-1246	2.7869	0.00031268	0.21825
hsa-let-7b-5p	2.5576	0.0081102	0.42226
hsa-miR-219a-5p	2.4948	0.010107	0.42226
hsa-miR-1268a	1.9735	0.0145	0.42226
hsa-miR-23c	1.95	0.019153	0.42226
hsa-miR-374a-5p	2.3427	0.019708	0.42226
hsa-miR-4516	2.5197	0.020561	0.42226
hsa-miR-184	1.8291	0.020736	0.42226
hsa-miR-101-3p	1.9111	0.021611	0.42226
hsa-let-7a-5p	2.2998	0.023618	0.42226
hsa-miR-3613-5p	1.6248	0.024505	0.42226
hsa-miR-25-3p	2.3439	0.0273	0.42226
hsa-miR-129-5p	1.8413	0.027587	0.42226
hsa-miR-640	1.6743	0.033276	0.42226
hsa-miR-575	1.722	0.035213	0.42226
hsa-miR-3928-3p	1.8586	0.036006	0.42226
hsa-miR-155-5p	1.796	0.036909	0.42226
hsa-miR-617	1.6748	0.038023	0.42226
hsa-miR-1286	1.7294	0.038491	0.42226
hsa-miR-944	1.5194	0.041288	0.42226
hsa-miR-1-5p	1.5383	0.041696	0.42226
hsa-miR-517c-3p + hsa-miR-519a-3p	1.6686	0.043067	0.42226
hsa-miR-323b-5p	1.6666	0.044566	0.42226
hsa-miR-15b-5p	2.5352	0.044714	0.42226
hsa-miR-4485-3p	1.8252	0.045798	0.42226
hsa-miR-363-3p	1.7328	0.046822	0.42226
hsa-miR-30a-3p	2.0541	0.047643	0.42226
hsa-let-7d-5p	1.6412	0.049826	0.42226

The table shows the miRNA with their Fold Change (FC), *p*-value (raw *p*-value), and the *p*-value corrected by FDR (*q*-value). T-test student was performed to obtain the raw *p*-value and FDR correction to obtain the *p*-value adjusted (*q*-value).

**TABLE 4:** miRNAs differently expressed according to clinical outcome at 3 months.

	FC	*p*-value	*q*-value
hsa-miR-451a	8.6692	0.021018	0.99993
hsa-miR-1246	2.238	0.021301	0.99993
hsa-miR-144-3p	4.9203	0.033741	0.99993
hsa-miR-630	2.7222	0.042347	0.99993

The table shows fold change (FC) of miRNAs comparing poor (mRS > 2) vs. good (mRS 0-2) clinical outcome at 3 months, the raw *p*-value (*p*-value), and the *p*-value adjusted by FDR (*q*-value). T-test student was performed to obtain the raw *p*-value and FDR correction to obtain the *p*-value adjusted (*q*-value).

In addition, ROC analysis was performed to study the specificity and the sensibility of each miRNA and the mean of a panel of selected miRNAs to predict WFNS and mRS at 3 months (Supplementary Table 2). Overall, the predictive accuracy improved when the ROC analysis was performed after the combination of the expression of 2 or 3 miRNAs together than when it was performed only with expression data from one miRNA or from all the selected miRNAs (Supplementary Table 2 and Figures 3B,C).

## Discussion

Gene expression studies have become a promising tool to identify biomarkers for diagnosis, prediction of treatment efficacy and estimation of prognosis after different forms of acute brain injury. In this exploratory study, we have analyzed the role of miRNAs as CSF biomarkers in SAH, which has been scarcely studied so far. We found that miRNAs were overexpressed in the CSF after suffering a SAH. Moreover, our data suggest a trend toward significant association between the overexpression of certain miRNAs and the presence of DCI, as well as worse mid-term clinical outcomes. Finally, network analysis allowed us to identify specific genetic pathways in which miRNAs may be involved in disease progression.

### MicroRNAs are overexpressed in the cerebrospinal fluid after subarachnoid hemorrhage

According to our results, miRNAs expression was significantly higher in the CSF samples in the acute setting of SAH (post-hemorrhagic day three) compared to healthy controls, an observation also made by other groups (Su et al., 2015; Powers et al., 2016; Stylli et al., 2017). In line with these previous studies, we also found an overexpression of miR-92a, -let-7, –204-5p, –223-3p, –337-5p, –451a, –489, –508-3p, –514-3p, –516-5p, –548, –599, –937, –1224-3p, and –1301 in SAH patients compared to the healthy patients (Su et al., 2015; Powers et al., 2016; Stylli et al., 2017), corroborating that miRNA can be detected in the CFS samples using NanoString technology and suggesting they might be a biomarker associated with the occurrence of SAH. Specifically, hsa-miR-451a, –223-3p, –548, –514, and -let-7a-5p are among the top 100 miRNAs differentially expressed in SAH compared with healthy patients. Our cluster analysis revealed that almost all the studied miRNAs were higher in the CSF after suffering SAH, but remarkably high levels were seen of hsa-miR-320e, hsa-miR-378e and hsa-miR-630, coincident with the findings of Bache et al. (2017) and Su et al. (2015). Among our differential-expressed miRNA, we found hsa-miR-320e and hsa-miR-451a that had already been described as potential biomarkers for early diagnosis of acute stroke (Stylli et al., 2017; Koopaei et al., 2021; Aldous et al., 2022) and hsa-miR-630 that had also been related with cancer disease (Wu et al., 2018; Liu et al., 2021) and SAH (Wang et al., 2014; Sun et al., 2019). Therefore, after an acute brain injury such as SAH, there seems to be an increased expression of specific miRNAs which might activate or inhibit signaling pathways responsible of DCI and clinical outcomes. Under such assumption, miRNAs could become valuable prognostic markers in the acute setting of SAH.

### MicroRNA expression seems different in patients with delayed cerebral ischemia

Furthermore, our findings suggest that miRNA expression might be different in those SAH patients who developed DCI compared with those who did not suffer such complication. Our data showed that hsa-miR-320e and hsa-miR-451a were overexpressed in patients with DCI compared to those patients without DCI, although differences were not statistically significant. Stylli et al. (2017) investigated miRNAs in 10 SAH patients with cerebral vasospasm and 10 SAH patients without, and identified miR-27a-3p, –516a-5p, –566, and –1197 as potential biomarkers for cerebral vasospasm but not for DCI (Stylli et al., 2017); yet none of them were found in our series. Another study by Lu et al. (2017) compared the expression of miRNAs between patients with and without DCI and found several miRNAs differentially expressed between these groups, including miR-1268 and miR-324 (Lu et al., 2017), which are in the top 25 miRNAs differentially expressed in our database. They also found miR-574, –339-5p, and –132, which are among the top 100 miRNAs in our patients with DCI. Su et al. (2015) showed that miR-324 was upregulated in SAH patients when compared to the healthy patients, but without statistically significant differences between DCI and non-DCI groups.

Bache et al. (2017) found increased levels of miR-10b-3p, 21-5p, –132-3p, –146a-5p, 193a-5p, and –221-3p and a relative decrease in miR-208a-3p, –490-3p, –520h, –553, and –643 in patients with DCI. Similar results were obtained in our database, except for miR-221-3p, which has been found to decrease in patients with DCI, and miR-21-5p, –132-3p, –146a-5p and 193a-5p, which were not among the positive miRNAs obtained after normalization of our data expression and therefore were not analyzed in the DCI group. Although we obtained similar results to the ones reported by Bache et al. (2017), the differences were not statistically significant in our samples.

### MicroRNAs may interact with several key pathways in subarachnoid hemorrhage

Another intriguing finding of our research is that miRNAs may interact with several key pathways involved in SAH pathophisiology. Findings from enrichment analysis with the KEGG and Reactome database showed that miRNAs were involved in the regulation of TGF-β pathway, among others. TGF-β is involved in a vast number of interactions and can have many roles depending on the cellular context. For instance, TGF-β1 increases after neurological damage in stroke and is a powerful angiogenic regulator (Krupinski et al., 1996). Since miRNAs involved in the inhibition of TGF-β were upregulated in our patients without DCI, we hypothesized that these miRNAs might be downregulating TGF-β, thus affecting in different pathways which may determine clinical outcomes. However, there is controversy about the effects of TGF-β levels. TGF-β1 modulates microglial phenotype and promotes recovery after intracerebral hemorrhage (ICH) (Taylor et al., 2017). Taylor et al. observed that patients with high levels of TGF-β in plasma had better outcomes 90 days after ICH, defending the role of TGF-β1 in functional recovery from ICH (Taylor et al., 2017). Contrary, in SAH patients high levels of TGF-β might be related to poor outcomes and vasospasm (Dietmann et al., 2012; Zhao et al., 2019). TGF-β also plays a function in various neurogenic processes, including the formation and elongation of axons (Abe et al., 1996), and initiation of neuronal migration (Yi et al., 2010). In addition, miR-324, –488, –410, and –499 miRNAs have been related to the regulation of MAPK, ERK, CREB and SMAD4, kinases and transcription factors downstream of TGF-β pathway (Lhuillier and Dryer, 2000; Derynck and Zhang, 2003), confirming the role of these miRNAs regulating these pathways.

A second pathway related to the three miRNAs (miR-324-5p, –488-3p, and –410-3p) obtained in our enrichment analysis is focal adhesion. Molist et al. (2020) described miRNA-324-5p as an inhibitor of alpha9-Integrin (ITGA9), which mediates interactions between adhesion molecules on adjacent cells and/or the extracellular matrix (ECM) (Calderwood, 2004; Carbon and Mungall, 2018). Dai et al. (2019) found that miR-488-3p regulates mitigation by inhibiting the Wnt signaling pathway, which is involved in the formation, maturation and maintenance of the BBB (Gupta et al., 2021). This agrees with the Reactome enrichment analysis where miR-410-3p has been related to the Wnt pathway in miRTarBase database. Therefore, another pathway that might be regulated by the differentially-expressed miRNAs in DCI patients is BBB disruption.

Finally, a third pathway that might be regulated by the three miRNAs (miR-324-5p, –488-3p, and –410-3p) used in our enrichment analysis was the Rap1 signaling pathway. Rap1 controls cell-to-cell and cell-to-matrix interactions by regulating the function of integrins and other adhesion molecules in various cell types (Wojciak-Stothard and Ridley, 2002; Wilson and Ye, 2014; Ramos and Antonetti, 2017; Wong et al., 2018), which seems to play a key role in BBB. Therefore, the aforementioned miRNAs, might also be related to BBB formation, permeability and disruption.

Since recent studies have correlated DCI and global cerebral ischemia with alterations of BBB permeability (Yan et al., 2011; Sanelli et al., 2012, 2013; Ivanidze et al., 2015), all these miRNAs we identified as able to regulate BBB integrity would play a crucial role in the complex pathophysiology of DCI following SAH.

### MicroRNAs as biomarkers of clinical outcomes in subarachnoid hemorrhage

According to our results, the majority of miRNAs were overexpressed in the poor WFNS group. Moreover, hsa-miR-451a and miR-630 were overexpressed in patients with poor outcomes at three months, with a statistically significant difference. Interestingly, hsa-miR-610 is one of two miRNAs overexpressed in patients with both good WFNS at admission and good outcome at three months, suggesting it could serve as a biomarker of favorable prognosis. Conversely, among the miRNAs overexpressed in patients with poor outcome, we found hsa-let-7, hsa-miR-1246, and hsa-miR-630. Of note, Sun et al. (2019) found that miR-630 plays an important role in endothelial dysfunction after SAH which could explain the results seen in our sample.

### Technologies

NanoString is one of the novel technologies that allow detecting miRNAs in a degraded and a small quantity of sample. The assay detects miRNAs without the use of reverse transcription or amplification by using molecular barcodes. Therefore, it is easier and faster to validate miRNA biomarkers as compared to RNASeq or PCR-based platforms and the results are highly reproducible. It is a unique biomarker discovery and signature development tool that enables the collection of expression data in a short amount of time with minimal hands-on manipulation. Thus, it may be a good technique to use in our study, where the RNA might be degraded and the quantity of samples to extract the RNA might be small. In addition, this technique might be a good technique to apply to clinical use, in the future.

Gene expression studies are a useful tool to identify biomarkers that are used to make diagnoses and predict treatment efficacy and disease prognosis. There are different technologies to study gene expression, such as RNA sequencing (RNAseq), microarrays, qPCR and NanoString (Prokopec et al., 2013; Chatterjee et al., 2015; Turnbull et al., 2020). RNAseq directly sequences and quantifies the number of mRNA molecules in the entire transcriptome. This makes it a powerful tool for detecting novel transcripts, gene fusions, single nucleotide variants, indels (small insertions and deletions), and other changes that arrays are not able to detect. RNAseq is highly sensitive and can be used to detect rare and low-abundance transcripts. However, high-quality RNA is needed, with a suggested RNA integrity number (RIN) over 8 and also requires a minimum of 1 μg total RNA, which are larger samples compared with NanoString. In exchange, with NanoString technology, high-quality RNA is not required. It is, therefore, ideal for scenarios where only poor-quality RNA is available, including biological fluids. Smaller amounts of starting material are needed compared to RNAseq: 100 ng of total RNA to NanoString compared to the 1ug of total RNA to RNAseq or >130 ng to microarrays. On the other hand, NanoString is more reproducible than qPCR, which needs more replicates due to their variance in the results. Additionally, sample preparation is the greatest source of error in any application. The multiple steps from sample collection, RNA extraction, reverse transcription, and data acquisition all provide opportunities for the introduction of error. In this sense, NanoString has the simplest preparation because it does not require reverse transcription, thereby reducing the likelihood of introducing technical variation. In addition, the NanoString platform has improved the detection of low-expression RNA compared to microarrays. Altogether, NanoString technology might be interesting to study the gene expression of miRNA in CSF.

## Limitations

In our dataset, only patients who required implantation of an EVD were included, therefore the sample was characterized by an overrepresentation of patients with poorer neurological status (WFNS > 2) upon admission and higher modified Fisher grades (3–4). This may limit the extrapolation of the results to all patients suffering from SAH. Moreover, patients with ultra-early mortality (within the first week) were excluded from the study, given that no complete follow-up could have been obtained; still, within the selected sample, mortality was only seen in the non-DCI group (mean mortality time-point on day + 10). This observation could denote a bias by which early mortality precluded from detection of DCI. Besides, regarding the methodology employed, a well-known limitation of cluster analysis is the performance of multiple comparisons. Nonetheless, to minimize the aleatory error, we corrected the significance testing with FDR. In addition, further experiments would be necessary to validate the selected miRNAs with alternative platforms, such as RT-qPCR.

## Conclusion

In this exploratory study, we have demonstrated that miRNAs can be studied in CSF in a cohort of SAH patients using NanoString technology. miRNAs seem to be overexpressed in the acute stage of SAH and presumably might play regulatory functions of pathophysiological pathways, such as those related with TGF-β (inflammation) and integrins (BBB disruption). The differential expression of specific miRNAs according to clinical onset, DCI development, and functional outcomes deserves further study in larger samples to reinforce their potential utility as prognostic and diagnostic biomarkers in SAH patients.

## Data availability statement

The original contributions presented in this study are publicly available. This data can be found here: https://www.ncbi.nlm.nih.gov/geo/query/acc.cgi?acc=GSE211002.

## Author contributions

RT, SA, and LP: conception and design. JH, LR, LL, DS, NR, RM, XS, SA, RT, and LP: data collection. LP, JH, LR, SA, and RT: analysis and interpretation of data. LP, JH, LR, AR-H, JE, SA, and RT: drafting the article. RT, SA, AR-H, and JE: revising it critically for important intellectual content. All authors final approval of the version to be published.

## Footnotes

1
https://www.mirnet.ca/

2
http://userver.bio.uniroma1.it/apps/mienturnet/

3
http://www.rproject.org/

4
www.metaboanalyst.ca
