# Supplementary material for: MicroRNA cerebrospinal fluid profile during the early brain injury period as a biomarker in subarachnoid hemorrhage patients
Source: Front Cell Neurosci. 2022 Nov 25;16:1016814. doi: 10.3389/fncel.2022.1016814 (PMC9732100; doi:10.3389/fncel.2022.1016814)
Supplement: Supplementary file 1 [file Data_Sheet_1.docx]

Supplementary Material

1. **SUPPLEMENTARY TABLES AND FIGURES**
   1. Figures


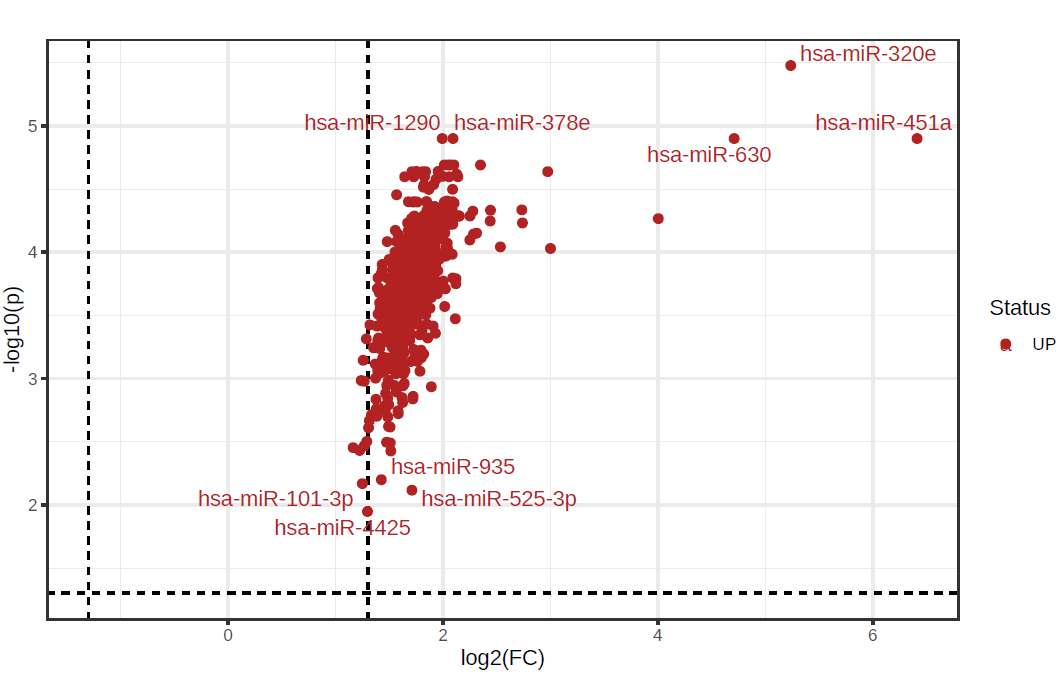


**Supplementary Figure S1**: Important features selected by volcano plot with fold change threshold >2 and t-tests threshold <0.1. The red circles represent features above the threshold. Both fold changes and p values are log-transformed. The further its position is away from the (0,0), the more significant the feature is. The comparison was SAH versus control samples. T-test student was performed to obtain the raw p-value.


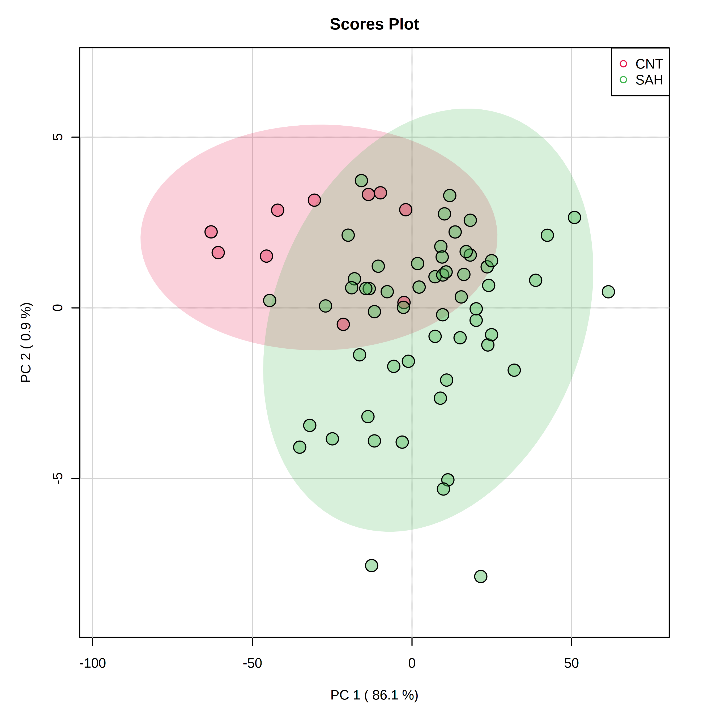


**Supplementary Figure S2:** Principal component analysis (PCA) of SAH (green area) and control (red area) samples according to the expression of miRNAs (green and red circles) included in the NanoString gene panel.


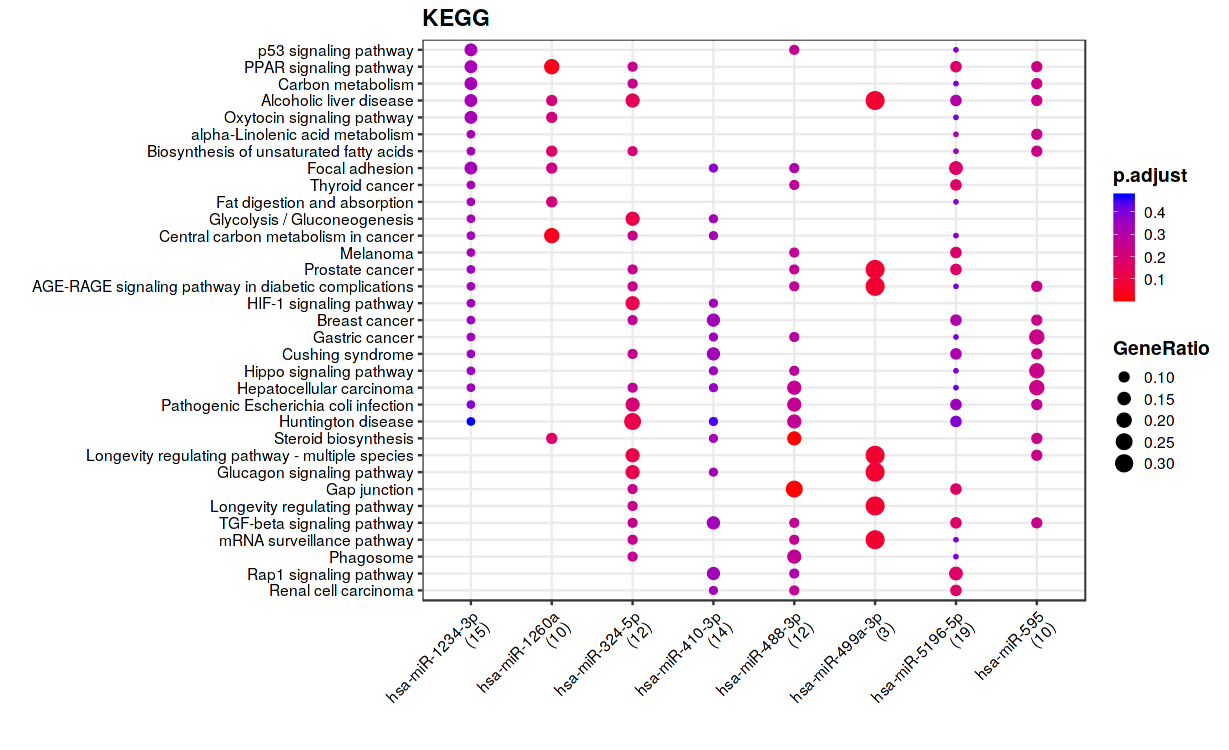


**Supplementary Figure S3:** Functional enrichment analysis performed with target genes of the most relevant miRNAs among 9 miRNAs of the DCI comparison, from miRTarBase database, using KEGG pathways. In x-axes are the miRNAs and in the y-axes are the pathways obtained with the genes that have interactions with the selected miRNA. The gene ratio is represented by the size of the dot and the color indicates the p-value adjusted by FDR.


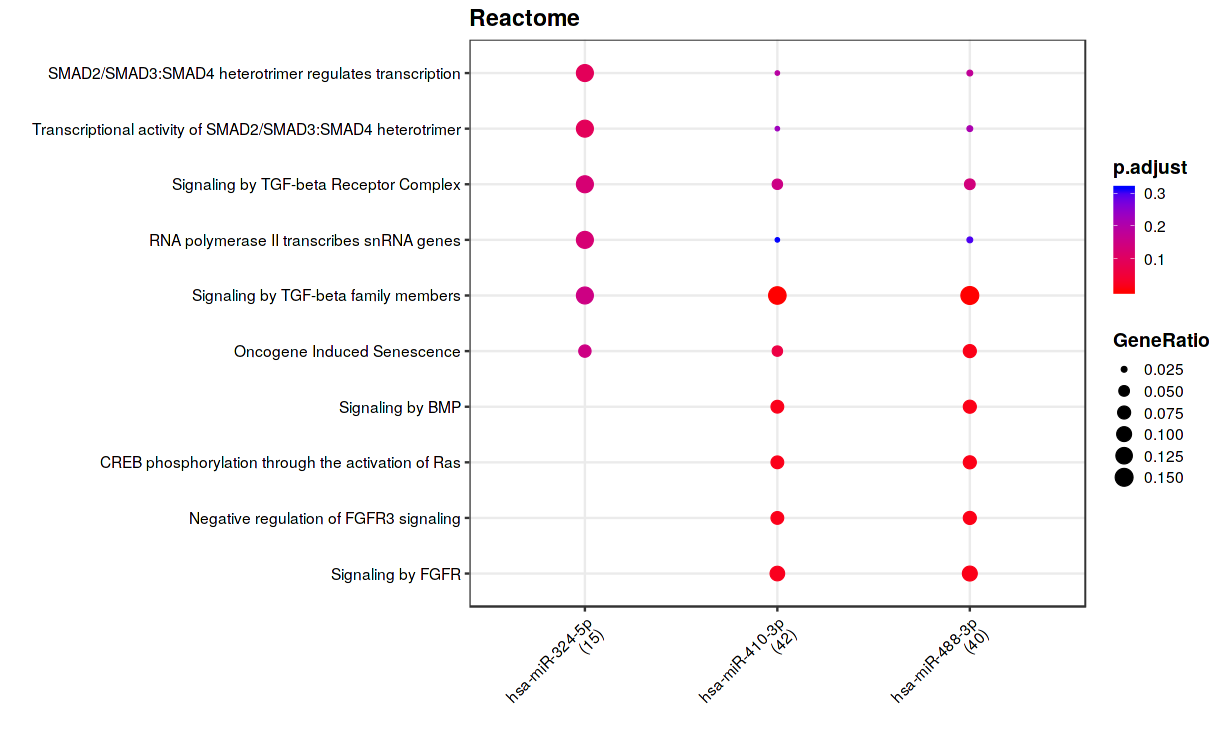


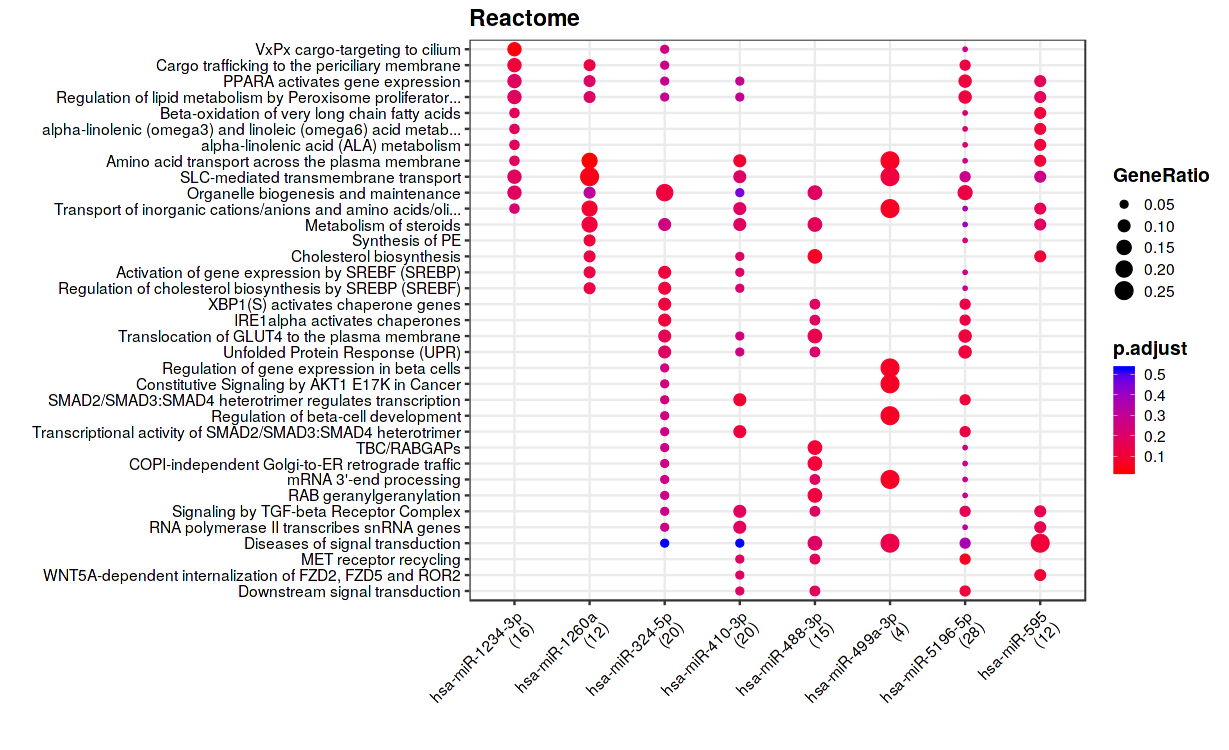


**Supplementary Figure S4:** Functional enrichment analysis performed with target genes of the most relevant miRNAs among 9 miRNAs of the DCI comparison, from Target Gene (top panel) and miRTarBase (bottom panel) database, using Reactome pathways. In x-axes are the miRNAs and in y-axes, the pathways are obtained with the genes that have interactions with the selected miRNA. The gene ratio is represented by the size of the dot and the color indicates the p-value adjusted by FDR.


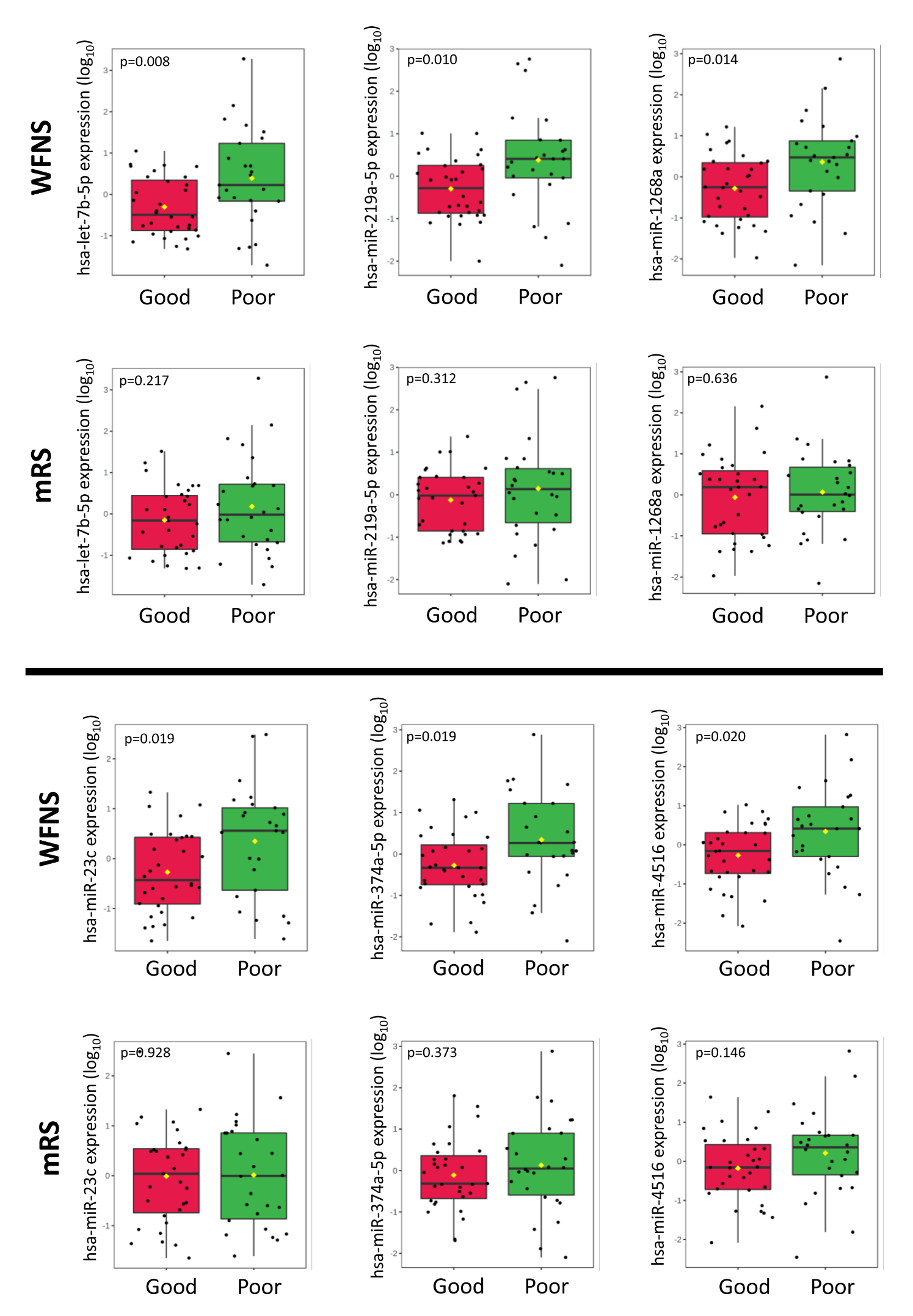

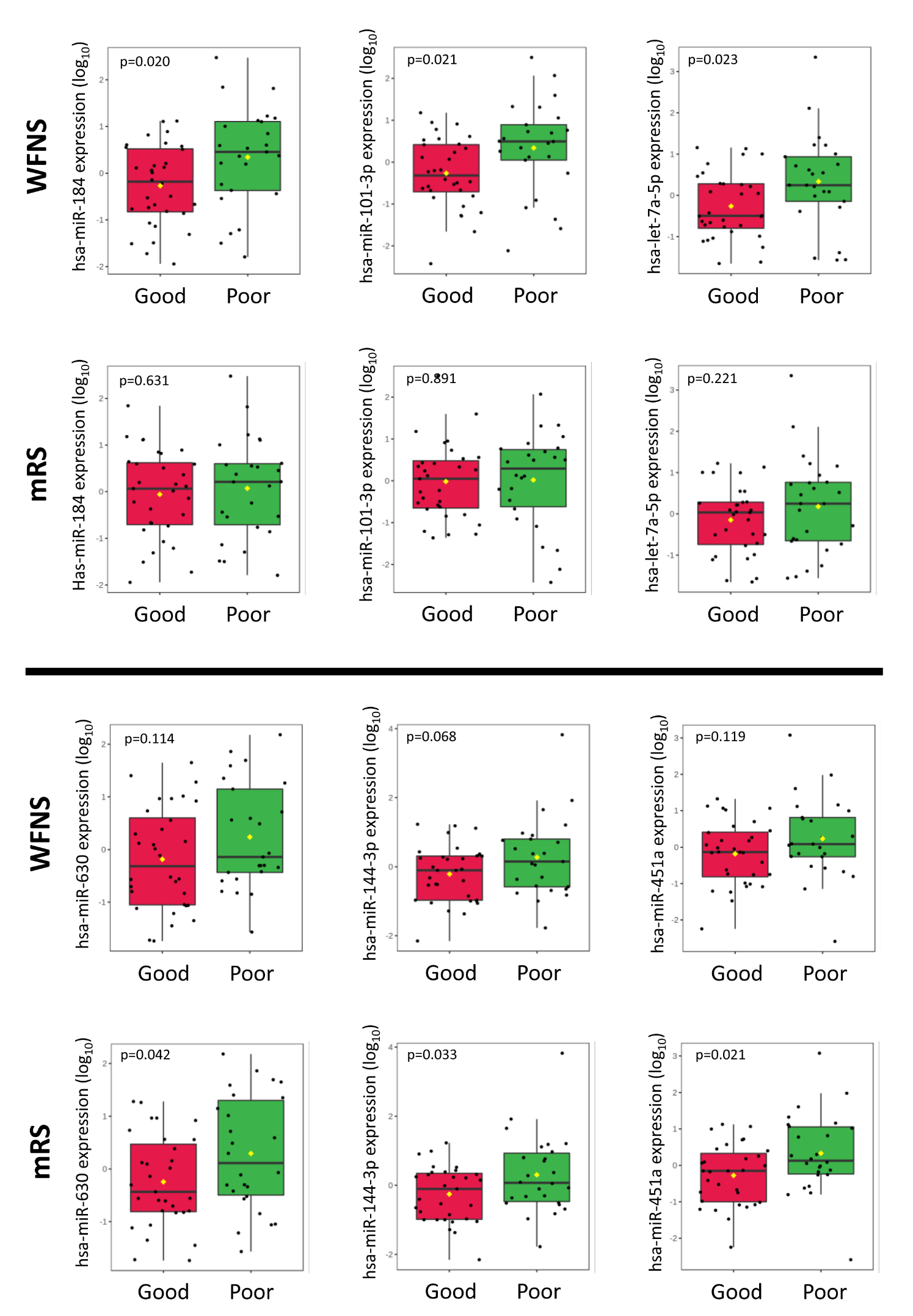


**Supplementary Figure S5:** Boxplots of the miRNA differentially expressed in WFNS and mRS comparison. The miRNA expression was compared between patients with a good (WFNS 1–3, red) or poor (WFNS 4–5, green) WFNS at the hospital admission (WFNS panels) and the good (mRS ≤ 2, red) or poor (mRS >2, green) outcome at 3 months (mRS panels), after SAH. P-value was obtained by t-test student, and the expression was transformed by log_10_.

- 1. Tables

**Supplementary Table S1:** Top 50 features identified by volcano plot comparing miRNA expression between SAH and control samples. In the table are shown the FC value (SAH versus control samples), the log_2_(FC), the p-value, and the p-value corrected by FDR (p-adjusted). T-test student was performed to obtain the raw p-value.

| miRNA | Fold Change | log_2_(FC) | p-value | p-adjusted |
| --- | --- | --- | --- | --- |
| hsa-miR-320e | 37.683 | 5.2358 | 4.76E-09 | 3.32E-06 |
| hsa-miR-378e | 4.2657 | 2.0928 | 4.95E-08 | 1.26E-05 |
| hsa-miR-630 | 26.163 | 4.7095 | 7.14E-08 | 1.26E-05 |
| hsa-miR-451a | 85.162 | 6.4121 | 7.39E-08 | 1.26E-05 |
| hsa-miR-1290 | 3.9797 | 1.9927 | 9.00E-08 | 1.26E-05 |
| hsa-miR-644a | 4.1372 | 2.0486 | 1.97E-07 | 2.03E-05 |
| hsa-miR-3144-3p | 4.0343 | 2.0123 | 2.61E-07 | 2.03E-05 |
| hsa-miR-1246 | 5.0940 | 2.3488 | 2.69E-07 | 2.03E-05 |
| hsa-miR-574-5p | 4.1870 | 2.0659 | 2.78E-07 | 2.03E-05 |
| hsa-miR-5196-5p | 4.2879 | 2.1003 | 2.91E-07 | 2.03E-05 |
| hsa-miR-2116-5p | 3.8749 | 1.9542 | 3.83E-07 | 2.29E-05 |
| hsa-miR-549a | 3.3712 | 1.7532 | 3.94E-07 | 2.29E-05 |
| hsa-miR-378h | 3.5733 | 1.8373 | 4.56E-07 | 2.30E-05 |
| hsa-miR-4536-5p | 3.5095 | 1.8113 | 4.78E-07 | 2.30E-05 |
| hsa-miR-223-3p | 7.8571 | 2.9740 | 4.95E-07 | 2.30E-05 |
| hsa-miR-548q | 3.2723 | 1.7103 | 5.26E-07 | 2.30E-05 |
| hsa-miR-1270 | 4.3680 | 2.1270 | 5.86E-07 | 2.41E-05 |
| hsa-miR-544a | 3.9928 | 1.9974 | 6.46E-07 | 2.51E-05 |
| hsa-miR-21-5p | 3.5491 | 1.8274 | 6.87E-07 | 2.52E-05 |
| hsa-miR-573 | 4.1607 | 2.0568 | 7.83E-07 | 2.52E-05 |
| hsa-miR-1185-1-3p | 4.4020 | 2.1381 | 7.89E-07 | 2.52E-05 |
| hsa-miR-4455 | 3.1219 | 1.6424 | 7.95E-07 | 2.52E-05 |
| hsa-miR-603 | 3.3106 | 1.7271 | 8.31E-07 | 2.52E-05 |
| hsa-miR-1285-5p | 3.8185 | 1.9330 | 9.12E-07 | 2.65E-05 |
| hsa-miR-491-5p | 3.7662 | 1.9131 | 1.04E-06 | 2.91E-05 |
| hsa-miR-888-5p | 3.5368 | 1.8225 | 1.09E-06 | 2.93E-05 |
| hsa-miR-1297 | 3.6048 | 1.8499 | 1.18E-06 | 3.04E-05 |
| hsa-miR-548ar-5p | 3.5241 | 1.8173 | 1.22E-06 | 3.05E-05 |
| hsa-miR-30e-5p | 4.2532 | 2.0886 | 1.32E-06 | 3.17E-05 |
| hsa-miR-147a | 3.6492 | 1.8676 | 1.37E-06 | 3.18E-05 |
| hsa-miR-1253 | 2.9642 | 1.5676 | 1.56E-06 | 3.50E-05 |
| hsa-miR-548e-5p | 4.1038 | 2.0369 | 1.80E-06 | 3.92E-05 |
| hsa-miR-411-5p | 3.5949 | 1.8459 | 1.91E-06 | 3.97E-05 |
| hsa-miR-590-5p | 3.2002 | 1.6781 | 2.06E-06 | 3.97E-05 |
| hsa-miR-519d-3p | 3.5933 | 1.8453 | 2.09E-06 | 3.97E-05 |
| hsa-miR-1245b-5p | 4.2477 | 2.0867 | 2.10E-06 | 3.97E-05 |
| hsa-miR-640 | 3.3272 | 1.7343 | 2.11E-06 | 3.97E-05 |
| hsa-miR-32-5p | 3.3857 | 1.7594 | 2.18E-06 | 4.00E-05 |
| hsa-miR-199b-5p | 4.0355 | 2.0128 | 2.25E-06 | 4.00E-05 |
| hsa-miR-548n | 3.2974 | 1.7213 | 2.29E-06 | 4.00E-05 |
| hsa-miR-361-5p | 4.2972 | 2.1034 | 2.40E-06 | 4.08E-05 |
| hsa-miR-548o-3p+hsa-miR-548ah-3p+hsa-miR-548av-3p | 4.0745 | 2.0266 | 2.61E-06 | 4.30E-05 |
| hsa-miR-570-3p | 3.6767 | 1.8784 | 2.65E-06 | 4.30E-05 |
| hsa-miR-548j-5p | 3.7965 | 1.9247 | 2.74E-06 | 4.34E-05 |
| hsa-miR-1203 | 3.7479 | 1.9061 | 2.90E-06 | 4.50E-05 |
| hsa-miR-15b-5p | 6.6490 | 2.7331 | 3.04E-06 | 4.61E-05 |
| hsa-miR-651-5p | 3.6067 | 1.8507 | 3.22E-06 | 4.64E-05 |
| hsa-let-7a-5p | 5.4324 | 2.4416 | 3.28E-06 | 4.64E-05 |
| hsa-miR-128-1-5p | 3.7145 | 1.8932 | 3.37E-06 | 4.64E-05 |
| hsa-miR-612 | 3.9725 | 1.9901 | 3.44E-06 | 4.64E-05 |

**Supplementary Table S2:** ROC analysis results. The table shows the miRNA or mean of three or more miRNAs, the Youden Index, the AUC value and its confidential interval (95%) and the p-value. The selected miRNAs were those that were differentially expressed in DCI-, WFNS- and modified Rankin Scale (mRS) at 3 months comparisons, and the mean of the expression of two or three firsts miRNAs or all selected miRNAs in each comparison.

| **Comparison** | **miRNA** | **Youden index** | **AUC (confidential interval)** | **p-value** |
| --- | --- | --- | --- | --- |
| **DCI** | hsa-miR-190b-5p | 0.514 | 0.747 (0.586, 0.907) | 0.020 |
|  | hsa-miR-5196-5p | 0.556 | 0.735 (0.565, 0.905) | 0.026 |
|  | hsa-miR-499a-3p | 0.507 | 0.738 (0.560, 0.917) | 0.024 |
|  | hsa-miR-595 | 0.410 | 0.706 (0.512, 0.900) | 0.051 |
|  | hsa-miR-410-3p | 0.382 | 0.685 (0.478, 0.893) | 0.080 |
|  | hsa-miR-324-5p | 0.417 | 0.726 (0.565, 0.886) | 0.033 |
|  | hsa-miR-488-3p | 0.424 | 0.683 80.481, 0.884) | 0.084 |
|  | hsa-miR-1234-3p | 0.431 | 0.671 80.445, 0.897) | 0.105 |
|  | hsa-miR-1260a | 0.382 | 0.681 (0.462, 0.899) | 0.088 |
|  | mean miR-5196-5p, miR-190b-5p and miR-499a-3p | 0.556 | 0.757 (0.598, 0.916) | 0.015 |
|  | mean 9 selected miRNAs | 0.431 | 0.725 (0.546, 0.907) | 0.032 |
| **WFNS** | hsa-miR-1246 | 0.581 | 0.789 (0.660, 0.917) | 0.000 |
|  | hsa-let-7b-5p | 0.354 | 0.680 (0.530, 0.830) | 0.021 |
|  | hsa-miR-219a-5p | 0.354 | 0.681 (0.532, 0.831) | 0.020 |
|  | hsa-miR-1268a | 0.381 | 0.690 (0.545, 0.835) | 0.015 |
|  | hsa-miR-23c | 0.475 | 0.679 (0.528, 0.829) | 0.021 |
|  | hsa-miR-374a-5p | 0.354 | 0.674 (0.528, 0.820) | 0.025 |
|  | hsa-miR-4516 | 0.341 | 0.672 (0.527, 0.818) | 0.026 |
|  | hsa-miR-184 | 0.368 | 0.679 (0.533, 0.825) | 0.021 |
|  | hsa-miR-101-3p | 0.376 | 0.680 (0.531, 0.829) | 0.021 |
|  | hsa-let-7a-5p | 0.403 | 0.674 (0.525, 0.822) | 0.025 |
|  | mean miR-1246, let-7b-5p and miR-219a-5p | 0.434 | 0.741 (0.606, 0.876) | 0.002 |
|  | mean 10 selected miRNAs | 0.403 | 0.713 (0.571, 0.854) | 0.006 |
| **mRS at 3 months** | hsa-miR-451a | 0.288 | 0.675 (0.534, 0.816) | 0.024 |
|  | hsa-miR-1246 | 0.344 | 0.672 (0.529, 0.815) | 0.026 |
|  | hsa-miR-144-3p | 0.288 | 0.649 (0.504, 0.794) | 0.055 |
|  | hsa-miR-630 | 0.282 | 0.649 (0.502, 0.796) | 0.055 |
|  | mean miR-1246 and miR-451a | 0.350 | 0.684 (0.544, 0.824) | 0.018 |
|  | mean 4 selected miRNAs | 0.311 | 0.680 (0.539, 0.821) | 0.020 |
